# Evaluations of UltraiQ software for objective ultrasound image quality assessment using images from a commercial scanner

**DOI:** 10.1002/acm2.12255

**Published:** 2018-01-16

**Authors:** Zaiyang Long, Donald J Tradup, Scott F Stekel, Krzysztof R Gorny, Nicholas J Hangiandreou

**Affiliations:** ^1^ Department of Radiology Mayo Clinic Rochester MN USA

**Keywords:** image quality, objective assessment, UltraiQ, ultrasound

## Abstract

We evaluated a commercially available software package that uses B‐mode images to semi‐automatically measure quantitative metrics of ultrasound image quality, such as contrast response, depth of penetration (DOP), and spatial resolution (lateral, axial, and elevational). Since measurement of elevational resolution is not a part of the software package, we achieved it by acquiring phantom images with transducers tilted at 45 degrees relative to the phantom. Each measurement was assessed in terms of measurement stability, sensitivity, repeatability, and semi‐automated measurement success rate. All assessments were performed on a GE Logiq E9 ultrasound system with linear (9L or 11L), curved (C1‐5), and sector (S1‐5) transducers, using a CIRS model 040GSE phantom. In stability tests, the measurements of contrast, DOP, and spatial resolution remained within a ±10% variation threshold in 90%, 100%, and 69% of cases, respectively. In sensitivity tests, contrast, DOP, and spatial resolution measurements followed the expected behavior in 100%, 100%, and 72% of cases, respectively. In repeatability testing, intra‐ and inter‐individual coefficients of variations were equal to or less than 3.2%, 1.3%, and 4.4% for contrast, DOP, and spatial resolution (lateral and axial), respectively. The coefficients of variation corresponding to the elevational resolution test were all within 9.5%. Overall, in our assessment, the evaluated package performed well for objective and quantitative assessment of the above‐mentioned image qualities under well‐controlled acquisition conditions. We are finding it to be useful for various clinical ultrasound applications including performance comparison between scanners from different vendors.

## 1 INTRODUCTION

Computer‐based tools for quantitative assessment of ultrasound image quality are valuable for ultrasound system purchase evaluation, acceptance testing, and image optimization. Thus, many efforts have been devoted to develop a set of algorithms for automated or semi‐automated evaluation of B‐mode image quality metrics in the past. For example, Rownd et al. reported an automated analysis process for testing the detectability of various contrast targets to represent the combined effects of axial, lateral and elevational spatial resolution.^1^ Gibson et al. developed a suite of computer programs to measure axial and lateral spatial resolution, penetration and sensitivity, which were demonstrated to have better reproducibility than manual and visual methods.^2^ Thijssen et al. reported and implemented a test protocol including a series of quantitative measurements of overall dynamic range, sensitivity, contrast resolution, and spatial resolution.^3^ Other methods for quantitatively assessing metrics such as resolution integral, depth of penetration (DOP), entropy, correlation, clutter, and spherical target detection have also been proposed.^4^, ^5^, ^6^, ^7^, ^8^, ^9^

However, most of the above methods were custom developed for in‐house use; therefore, their widespread use was limited. A readily available commercial tool that is subject to regular vendor‐provided maintenance and upgrades, and does not require additional programming input by the user, would be very beneficial to a wide audience of medical physicists for routine use in the clinical environment. Recently, a commercially available software package, UltraiQ (Cablon Medical B.V., Leusden, The Netherlands), has become available as a candidate for quantitative evaluation of B‐mode image quality using images acquired in compatible commercial phantoms (e.g., Model 404 from Gammex Inc., ATS 539 from the ATS Laboratories, or Model 040 from CIRS Inc.). The package delivers a variety of automated performance measurements that include DOP, contrast response, spatial resolution, geometric accuracy, and loss of element analysis. For our practice, we initially determined that the most useful UltraiQ measurements were contrast response, spatial resolution (lateral, axial, and elevational), and DOP. The software attempts to automate these measurements by locating the relevant phantom targets or image regions without operator input, which should reduce the measurement time. Additionally, if automation fails, the operator can provide manual input to complete the analysis.

Overall, the aim of this study was to use images from a clinical ultrasound scanner to evaluate the clinical utility of the UltraiQ package for quantitative assessment of contrast response, spatial resolution and DOP, considering measurement stability, sensitivity, repeatability, and success rate for the semi‐automated measurement process.

## 2 MATERIALS AND METHODS

All UltraiQ measurements were made with images obtained with a GE Logiq E9 ultrasound system (GE Healthcare, Milwaukee, WI, USA) coupled with linear (9L or 11L), curved (C1‐5) and sector (S1‐5) transducers. (The 11L was used only for the DOP measurement assessment, as the 9L did not allow all of the desired control manipulations to be made without pixel value saturation.) Each UltraiQ measurement was evaluated with a transducer of each type (linear, curved, or sector) using a CIRS model 040GSE phantom (CIRS Inc., Norfolk, VA, USA). The 0.7 dB/cm/MHz regions of the CIRS phantom were used in all cases except for one of the DOP sensitivity experiments, where we purposefully changed to the 0.5 dB/cm/MHz background. Time gain compensation slide potentiometers were in their right‐most position for consistency, and a single focal zone at the bottom of the image was used for all acquisitions. Prior to UltraiQ processing, all images were analyzed to assure the absence of pixel saturation using an in‐house MATLAB program (MATLAB R2013b, MathWorks, Natick, MA, USA). Six sets of images or image pairs were acquired and averaged for each measurement.

The following UltraiQ measurements were evaluated:

1. **Contrast response** (in unit of pixel gray levels/dB) measured by analyzing pixel gray level value differences from three or more cylindrical targets in the phantom with known differences in echogenicity. Before computing contrast response, the program analyzes the grayscale ramp in the image to account for nonlinearities in the selected image gray map.^3^
2. **DOP** defined as the depth at which the SNR falls to a predefined threshold value. The UltraiQ program uses the IEC algorithm^10^ to compute SNR from a pair of images (one of a plain gel region of the phantom and the other in air) with the same acquisition parameters. It then defines DOP in units of “cm” as the depth at which SNR falls to 1.
3. **Spatial resolution** in the lateral, axial, and elevational directions measured from trans‐axial images of a column of nylon filament (or “pin”) targets. The lateral spatial resolution is taken to be the lateral dimension of each pin at which the pixel value falls to 6 dB below the peak pixel value in the pin. The relationship between pixel value and dB is defined through an evaluation of contrast response as described above. The axial spatial resolution is measured in a similar fashion as the lateral resolution. The software is *not* inherently designed to assess elevational spatial resolution, but we attempted this measurement by acquiring images with the scan plane oriented to intersect the column of pins at a 45‐degree angle,^11^, ^12^ and then measuring lateral pin dimensions as described above. Spatial resolution can be assessed as a function of pin depth. However, the results of the stability, sensitivity, and repeatability assessments of this measurement are reported for the single pin closest to the observed lateral or elevational focal point. If no clear focal depth was observed, the single pin closest to half of the DOP was used. Pin depths used for stability, sensitivity and repeatability assessments of lateral/axial, and elevational resolution measurements were 4 and 2 cm, 5 and 4 cm, 7 and 5 cm, for the 9L, C1‐5, and S1‐5, respectively.

Each of the three measurements described above were assessed in terms of stability, sensitivity, repeatability, and success rate of semi‐automated measurements.

*Stability* tested the undesired measurement variation from a baseline value when image parameters *not* fundamentally related to each measurement were changed. For example, UltraiQ measurements should not vary in response to changes in overall gain or gray map. Tables 1, 2, 3 detail the baseline values for each measurement and the image parameter changes used for stability and sensitivity assessments. Stability results were defined as the following: for variation related to an image parameter change (e.g., increased overall gain), deviation was defined as the percent error from the baseline value for that parameter. A threshold of ±10% was judged to be an acceptable level of variability.

**Table 1:** Imaging parameters for stability and sensitivity evaluation of the contrast response measurement. Baseline parameters list the nominal, starting point scan parameters. Stability and sensitivity parameters match the baseline parameters except for the indicated changes

Contrast response test	9L	C1‐5	S1‐5
Baseline			
Transmit frequency	7 MHz	4 MHz	3 MHz
Power	60%	60%	60%
Gain	85 dB	65 dB	60 dB
Dynamic range	69 dB	84 dB	84 dB
Gray map	D	D	D
Phantom background	0.7 dB/cm/MHz gel	0.7 dB/cm/MHz gel	0.7 dB/cm/MHz gel
Stability			
Changed gain/power	80/60%, 85/100%, 85/30%, 90/60%	60/60%, 65/100%, 65/30%, 70/60%	55/60%, 60/100%, 60/30%, 65/60%
Changed gray map	A and J	A and J	A and J
Changed frequency	9 MHz	5 MHz	4 MHz
Sensitivity			
Changed dynamic range	48 and 90 dB	75 and 96 dB	76 and 96 dB

**Table 2:** Imaging parameters for stability and sensitivity evaluation of the depth of penetration (DOP) measurement. Baseline parameters list the starting point scan parameters. Stability and sensitivity parameters match the baseline parameters except for the indicated changes. The second sensitivity test used the baseline scan parameters but with the 0.5 dB/cm/MHz gel region of the CIRS phantom

Depth of penetration test	11L	C1‐5	S1‐5
Baseline			
Transmit frequency	11 MHz	5 MHz	4 MHz
Power	100%	100%	100%
Gain	80 dB	85 dB	75 dB
Dynamic range	90 dB	90 dB	90 dB
Gray map	D	D	D
Phantom background	0.7 dB/cm/MHz gel	0.7 dB/cm/MHz gel	0.7 dB/cm/MHz gel
Stability			
Changed gain/dynamic range	75/90, 80/84, 80/96, 85/90	80/90, 85/84, 85/96, 90/90	70/90, 75/84, 75/96, 80/90
Changed gray map	A and J	A and J	A and J
Sensitivity			
Changed power	50% and 20%	50% and 20%	50% and 20%
Changed phantom background	0.5 dB/cm/MHz gel	0.5 dB/cm/MHz gel	0.5 dB/cm/MHz gel

**Table 3:** Imaging parameters for stability and sensitivity evaluation of the spatial resolution measurements. Baseline parameters list the starting point scan parameters. Stability and sensitivity parameters match the baseline parameters except for the indicated changes

Spatial resolution test	9L	C1‐5	S1‐5
Baseline			
Transmit frequency	7 MHz	4 MHz	3 MHz
Power	100%	100%	100%
Gain	85 dB	63 dB	60 dB
Dynamic range	90 dB	90 dB	90 dB
Gray map	D	D	D
Phantom background	0.7 dB/cm/MHz gel	0.7 dB/cm/MHz gel	0.7 dB/cm/MHz gel
Stability			
Changed gain/dynamic range	80/90, 85/84, 85/96, 90/90	58/90, 63/84, 63/96, 68/90	55/90, 60/84, 60/96, 65/90
Changed gray map	A and J	A and J	A and J
Sensitivity			
Changed transmit frequency	5 and 9 MHz	2 and 5 MHz	2 and 4 MHz

*Sensitivity* verified the expected measurement variation or trend when image parameters that *are* related to each measurement were changed. For example, DOP is expected to decrease as power output decreases, in‐plane spatial resolution is expected to improve as transmit frequency is increased, and contrast response is expected to change in response to dynamic range changes. For contrast response, a baseline dynamic range (DR) was changed to a higher and a lower value, and contrast response was plotted against 256/DR to assure a linear relationship. Sensitivity results were qualitatively assessed according to the predicted trend.

*Repeatability* tested the variations of each measurement made using a linear transducer, among four different operators and across two measurement sessions spanning a month. Scan experiences of the operators differed between 3 months and 10 years. Repeatability was represented by intra‐individual and inter‐individual coefficients of variation (CVs). Finally, the *success rate of semi‐automated measurement* for each measurement was determined.

## 3 RESULTS

**Figure 1:**
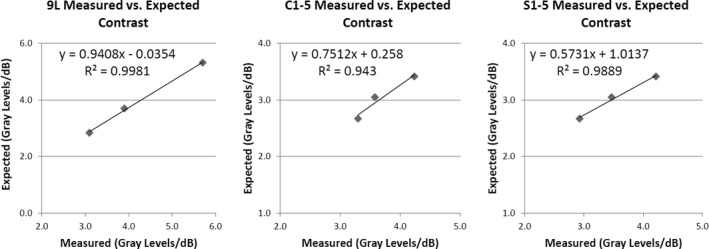
Sensitivity results for contrast response measured with 9L, C1‐5, and S1‐5 transducers. Three dynamic range values were used for each transducer as detailed in Table 1.

For the **contrast response** measurements, 19 of the 21 *stability* tests met the ±10% variability criteria. Parameter‐related variations were between 0.6% and 10.4% for 9L, between 1.7% and 4.7% (except 15.6% for gray map A) for C1‐5, and between 1.7% and 8.9% for S1‐5. *Sensitivity* test results are shown in Fig. 1. The measured contrast response for all transducers showed the expected linear correlations with respect to the predicted values equal to 256/DR (all *P* < 0.001). However, the slopes and intercepts of the best‐fit lines were different among the three transducers. *Repeatability* results showed intra‐individual CVs of 0%–3.2%. Inter‐individual CV was 3.2%. The *success rate of semi‐automated measurements* of contrast response was 0%, 44.4%, and 40.7%, for the 9L, C1‐5, and S1‐5 transducers, respectively. However, as previously mentioned, manual intervention resulted in valid measurements in all cases at the expense of increased measurement time.

**Figure 2:**
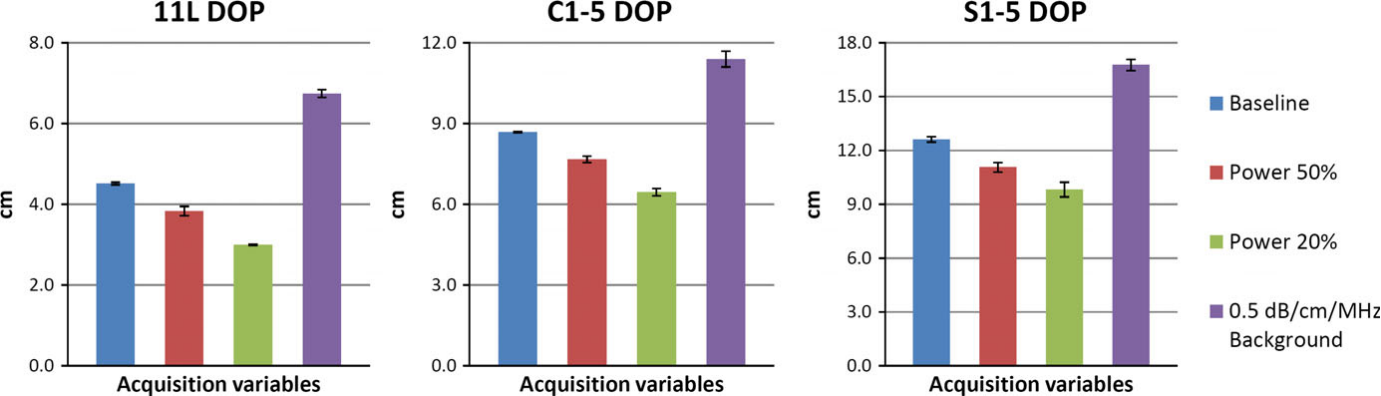
Sensitivity measurements of the depth of penetration (DOP) of an 11L, C1‐5, and S1‐5 transducer. Mean and standard deviation were obtained from six sets of phantom and in‐air image pairs. Specific power output values are detailed in Table 2.

For **DOP**, all of the 18 *stability* tests met the 10% variability criteria. Individual parameter‐related variations were between 2.8% and 8.5% for 11L, between 0.1% and 8.5% for C1‐5, and between 0.6% and 3.8% for S1‐5. *Sensitivity* test results shown in Fig. 2 revealed decreasing DOP with decreasing power output, and increased DOP in the less‐attenuating background compared with baseline, as expected. *Repeatability* results revealed intra‐individual CVs between 0.2% and 1.3%. Inter‐individual CV was 1.0%. The *success rate of semi‐automated measurements* of DOP was 100% for all three transducer models.

**Figure 3:**
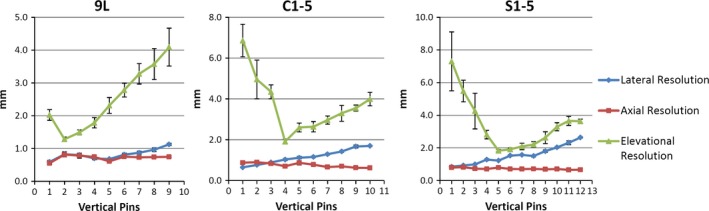
Baseline spatial resolution measurements versus pin depth for the 9L, C1‐5, and S1‐5 transducers. Mean and standard deviation were obtained from six sets of images.

Figure 3 depicts the lateral, axial, and elevational **spatial resolution** measurements for the three transducers as a function of pin depth in the baseline condition. The qualitative behavior of the individual measurements as a function of depth agrees with expectation: elevational resolution is worst and varies the most versus depth, with a distinct hourglass profile; axial resolution is best and remains roughly constant versus depth; lateral resolution is between the other two and demonstrates modest changes versus depth. Spatial resolution results will be reported across all resolution directions. For **spatial resolution**, 37 of the 54 *stability* tests met the ±10% variability criteria. Individual parameter‐related variations were between 1.4% and 20.3% for 9L, between 0.2% and 24.5% for C1‐5, and between 0% and 15.1% for S1‐5.

**Figure 4:**
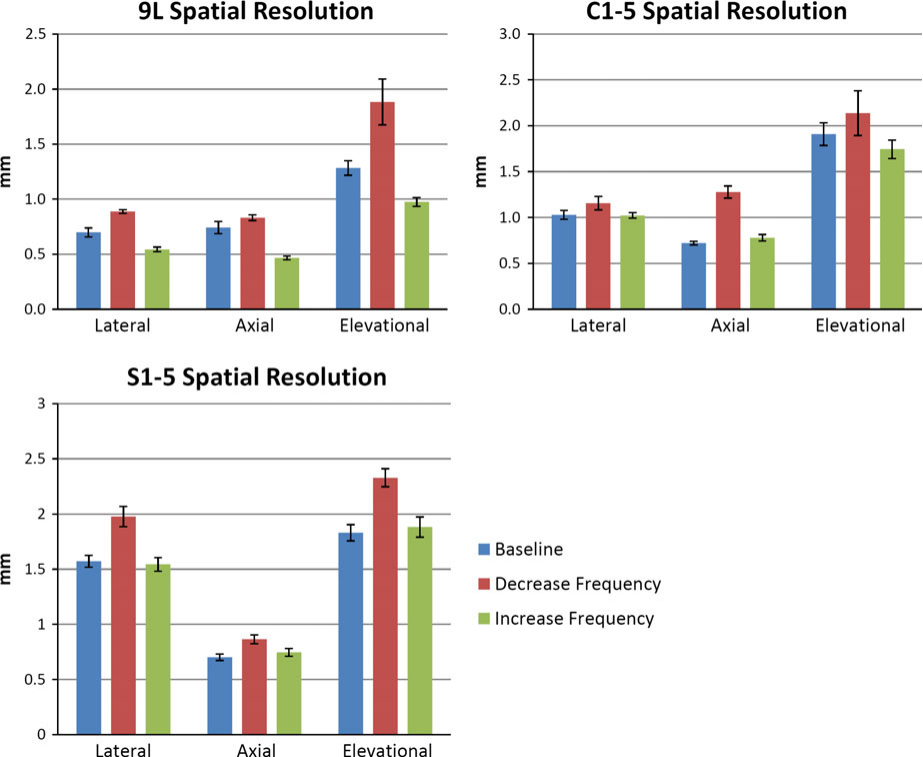
Sensitivity results for spatial resolution measured with 9L, C1‐5, and S1‐5 transducers. Specific frequency values are detailed in Table 3. Mean and standard deviation were obtained from six sets of images.

Figure 4 shows the *sensitivity* results for the spatial resolution measurements. Of the nine sets of individual spatial resolution measurements shown, all nine measurements showed worse resolution when transmit frequency was decreased, as expected. Four of the nine resolution measurements clearly improved in response to an increase in transmit frequency, while five remained relatively unchanged. These five tests involved transmit frequency increases of only 1 MHz. *Repeatability* results revealed intra‐individual CVs between 0% and 4.4%, except for a value of 9.5% for elevational resolution. Inter‐individual CV was 8.2%. The *success rate of semi‐automated measurements* of spatial resolution was 86.4%, 63.9%, and 58.6%, for the 9L, C1‐5, and S1‐5 transducers, respectively.

## 4 DISCUSSION

In this work, we evaluated the stability, sensitivity, repeatability, and success rate of semi‐automated measurements of contrast response, DOP, and spatial resolution performed using the UltraiQ software package. The measurement of **contrast response** exhibited excellent performance with respect to stability (90% of tests met our ±10% variability criteria), sensitivity (expected linear response versus 256/DR was seen in all cases), and repeatability (inter‐ and intra‐individual CVs were <= 3.2%). The variation in best‐fit slopes for the three transducers was initially surprising, but it was confirmed by the scanner vendor that contrast measurements for different probes may behave in this manner (personal communication with Dave Dubberstein, System Engineering Manager, GE Healthcare). The success rate of semi‐automatic analysis for the three probes was poor; however, as previously mentioned, manual intervention resulted in valid measurements in all cases at the expense of increased measurement time. For some applications, e.g., initial acceptance testing, an extended measurement time would be acceptable. Also, a new version of the UltraiQ software package paired with a specific custom phantom has recently been released, which is expected to improve the success of automated analysis of all measurements.

The **DOP** measurement exhibited the best overall performance for all four assessments. All tests of stability met the ±10% variability criteria. All expected sensitivity trends were observed. Inter‐individual CVs were <= 1.3% and intra‐individual CV was 1.0%. Success rate of semi‐automatic measurement was 100%.

For the **spatial resolution** measurements, the majority of the tests of stability met the ±10% variability criteria, but an appreciable number (31%) did not. In particular, certain changes in gray map, gain, or DR caused deviations in spatial resolution >10%. Therefore, we recommend keeping the set of parameters as consistent as possible for clinical measurements of spatial resolution. We also recommend using the most linear gray map available on each scanner, and adjusting overall gain so that the pin and contrast cylinder targets have relatively consistent gray levels. Expected sensitivity trends were observed in 13 of the 18 tests (72%), while no appreciable change was seen in the remaining 5. Expected spatial resolution changes were observed in all nine tests where frequency could be altered from the baseline by 2 MHz, but if frequency changes were limited by the scanner to 1 MHz, expected changes were seen in only four of the nine tests. Consideration of these stability and sensitivity results points to a limitation of our study design, namely that we are assuming (based on ultrasound imaging physics) that certain parameter changes should not affect a particular measurement or that other changes should affect the measurement. However, an actual imaging system may indeed exhibit paradoxical behavior with regard to scan parameter changes, for example, systems that do not lose image brightness when the power output control is reduced because an automatic increase in overall gain is *also* applied to compensate. It is not entirely clear that stability or sensitivity test results not agreeing with expected scanner behavior are due to a deficiency in the analysis software, or a true but unexpected behavior of the ultrasound scanner. Given that the majority of stability and sensitivity tests, 102 of 126 tests or 81%, do agree with the expected behavior, we hypothesize that the inconsistent results are more likely a result of unexpected scanner behavior rather than poor performance of the UltraiQ tool. However, system vendors are typically reluctant to discuss the details of specific engineering‐related issues.

This study limitation stems from our goal of testing the UltraiQ package using actual ultrasound images acquired on a commercial scanner, since that situation mimics actual clinical use of the tool. Future work will investigate artificially altering pixel values in ultrasound scanner images and then verifying that the relevant measurement varies in accordance with these alterations. Repeatability results were excellent for the lateral and axial spatial resolution measurement and worsened for the elevational spatial resolution measurement. The average success rate of semi‐automatic measurement was approximately 70%.

For elevational spatial resolution, Goldstein initially reported using the scan plane oriented at 45 degree to the ultrasound beam, such as scanning an inclined‐plane phantom, to acquire multiple scans in order to obtain the profile of the elevational resolution.^11^ Later, Skolnick proposed to simply orienting the scan plane to intersect the nylon filaments at 45 degree using regular flat‐surface commercially available phantoms, as an estimate of the elevational resolution.^12^ We used the “Skolnick” method to estimate the elevational resolution in this study. It should be noted that this method also partially reflects lateral resolution and the in‐plane beam width should be narrower than the elevational beam width to decrease the effect of the lateral resolution. Measurement of elevational resolution is not currently offered as part of the UltraiQ package suggesting that optimization of the algorithm to improve this measurement is possible. In our opinion, the lack of an elevational resolution measurement is a shortcoming of the program, as elevational resolution is a very important metric for performance evaluation.

Currently, UltraiQ only reports pixel saturation along with results for the DOP measurement. Pixel saturation is not analyzed for other UltraiQ measurements, and we consider this to be a major shortcoming. Users must be cautious and test for pixel saturation prior to image analysis with UltraiQ to guard against pixel saturation which can cause any of the measurements to be invalid. This is not a difficult test but does require pixel ROI tools on the ultrasound scanner, or another analysis program or programming environment. Given the typical 8‐bit‐per‐pixel limitation in DICOM images produced by commercial ultrasound scanners, we feel that saturation testing should be an integral part of any US scanner performance measurement tool.

The loss of element measurement in the software was not evaluated in this study. This is due to the fact that we use a custom uniformity test in our practice, based on qualitatively evaluating the median image of a clip using an in‐house liquid phantom which produces uncorrelated speckle patterns.^13^^.^

## 5 CONCLUSIONS

Despite the limitations in automatic measurement success and lack of pixel saturation evaluation, the UltraiQ software package appears to perform well for making an objective, quantitative assessment of B‐mode image contrast, DOP, and spatial resolution. We have found the tool to be very useful for testing scanner performance among the same scanner model, e.g., acceptance testing, where scan parameters can be made consistent. We have also used the software to compare performance of different scanner models, and here we recommend using the most linear gray map available on each scanner, and adjusting overall gain so that the pin and contrast cylinder targets have relatively consistent gray level.

## CONFLICT OF INTEREST

The authors have no conflict of interest to declare.
